# QTLs for stomatal and photosynthetic traits related to salinity tolerance in barley

**DOI:** 10.1186/s12864-016-3380-0

**Published:** 2017-01-03

**Authors:** Xiaohui Liu, Yun Fan, Michelle Mak, Mohammad Babla, Paul Holford, Feifei Wang, Guang Chen, Grace Scott, Gang Wang, Sergey Shabala, Meixue Zhou, Zhong-Hua Chen

**Affiliations:** 1 School of Science and Health, Hawkesbury Institute for the Environment, Western Sydney University, Penrith, NSW 2751 Australia; 2 School of Environmental Science and Engineering, Tianjin University, Tianjin, 300072 China; 3 School of Land and Food and Tasmanian Institute of Agriculture, University of Tasmania, Hobart, TAS 7249 Australia; 4 College of Agriculture and Biotechnology, Zhejiang University, Hangzhou, 310058 China

**Keywords:** Gas exchange, Guard cell, *Hordeum vulgare* L, QTL mapping, Stomatal regulation

## Abstract

**Background:**

Stomata regulate photosynthesis and transpiration, and these processes are critical for plant responses to abiotic stresses such as salinity. A barley double haploid population with 108 lines derived from a cross between CM72 (salt-tolerant) and Gairdner (salt-sensitive) was used to detect quantitative trait loci (QTLs) associated with stomatal and photosynthetic traits related to salinity tolerance.

**Results:**

A total of 11 significant QTLs (LOD > 3.0) and 11 tentative QTLs (2.5 < LOD < 3.0) were identified. These QTLs are distributed on all the seven chromosomes, except 5H and explain 9.5–17.3% of the phenotypic variation. QTLs for biomass, intercellular CO_2_ concentration, transpiration rate and stomatal conductance under control conditions co-localised together. A QTL for biomass also co-located with one for transpiration rate under salinity stress. A linkage was found between stomatal pore area and gas exchange. A QTL for salinity tolerance also co-localised with QTLs for grain yield and biomass on chromosome 3H. Based on the draft barley genome, the candidate genes for salinity tolerance at this locus are proposed.

**Conclusions:**

The lack of major QTLs for gas exchange and stomatal traits under control and saline conditions indicates a complex relationship between salinity and leaf gas exchange due to the fact that these complex quantitative traits are under the control of multiple genes.

**Electronic supplementary material:**

The online version of this article (doi:10.1186/s12864-016-3380-0) contains supplementary material, which is available to authorized users.

## Background

Soil salinity results from natural causes such as from soluble salts from rocks and oceanic salts carried in wind and rain as well as from increasing salinization of agricultural land due to irrigation and deforestation [1, 2]. Salinity is causing major global food security issues due to the large arable area that is now saline and not suitable for cropping; therefore, breeding salt tolerant crops has become a top priority. Genetic modification to produce transgenic plants containing novel genes or different expression levels of existing genes can improve plant salt tolerance [3]. However, salinity tolerance is controlled by multi-gene traits, where genes are expressed at a number of plant developmental stages in a highly tissue-specific manner. Genetic engineering of single genes has proven problematic for improving salt tolerance in crops [4], and it is unlikely that salt tolerance could be improved by manipulating the expression of only one or few genes [2, 5]. However, molecular breeding could be used to breed salt tolerant crops by exploiting existing genetic variation through direct selection or marker-assisted selection in conjunction with the use of quantitative trait loci (QTLs) for gene pyramiding.

Stomata are formed by two highly specialised guard cells, and some are surrounded by subsidiary cells in certain plant species like barley [6]. Stomata control the exchange of water vapour and CO_2_ between the leaf interior and the atmosphere, and serve as major gateways for CO_2_ influx into plants as well as transpirational water loss from plants [7–9]. Transpirational water loss through stomatal pores accounts for 70% of total water loss indicating their significance in water use [10]. The stomatal aperture is influenced by the plant and its environment [11]. Under saline conditions, plant cells lose water and reduce cell elongation for short-term osmotic adjustment and later build up cellular NaCl over a longer period [2, 12]. The accumulation of NaCl in plant cells, including stomatal guard cells, affects their function. Stomatal closure is one of the most immediate responses to salinity [2, 13, 14], and this response is believed to be crucial for minimising plant water loss under hyperosmotic conditions in their rhizosphere [15–17]. Reducing stomatal density is another way of optimising the balance between leaf water loss and CO_2_ assimilation. Halophytes, naturally salt-tolerant species, are capable of reducing stomatal density when grown under hypersaline conditions [18, 19]. The same effect has been observed in most tolerant barley varieties [20]. However, this strategy has a cost, as a reduction in stomata will reduce photosynthesis thereby reducing plant biomass and crop yield [21].

Stomatal and photosynthetic parameters, such as stomatal size and frequency, stomatal conductance, carbon assimilation, transpiration rate and water use efficiency, affect the grain yields of crops under stressed and non-stressed conditions [22, 23]. Genotypic variation in stomatal traits has been reported, but little is known about the genetic mechanisms behind these traits. A negative association between water loss and stomatal size was found in durum wheat [24], and while Wang and Clarke’s [25] growth-room experiment reported a positive correlation between stomatal density and the rate of water loss in excised wheat leaves. Stomatal paremeters (e.g., stomatal aperture, guard cell volume, aperture width and aperture width/length) were significantly different between salt-tolerant and salt-sensitive genotypes. Significant correlations have been found between stomatal traits, expression of slow anion channel genes and grain yield in salt-tolerant barley [26]. This suggests that stomatal traits may contribute to salinity tolerance in barley, but further study using genetically different populations is required.

Many studies on the salinity tolerance of plants focus on ionic relations, but there has been little research to determine the potential role of stomatal function in salinity tolerance. QTLs for gas exchange and stomatal parameters under greenhouse conditions or different stresses have been identified in *Arabidopsis* [27], rice [8, 28, 29], sunflower [30], and faba bean [31]. In barley, QTLs associated with net photosynthetic rate have been detected under drought stress [32] and without stress [33], and QTLs for stomatal conductance [33] and stomatal density [34] have been identified in barley grown without stress. However, to the best of our knowledge, QTLs for stomatal traits, especially stomatal aperture and guard cell and subsidiary cell geometry under salinity stress, have not been reported in plants.

In our recent work, we have explored stomatal and photosynthetic traits as potential selection criteria for plant salt tolerance [26]. Here, we measured stomatal and photosynthetic traits in a double haploid (DH) population of barley to identify significant QTLs. We hypothesised that stomatal traits are controlled by multiple genes and would result in multiple QTLs for salt tolerance in barley. Thus, the objectives of this study were to: (1) identify QTLs for stomatal and photosynthetic traits associated with salinity tolerance in barley, and (2) investigate the relationships between salinity tolerance and stomatal regulation through QTL mapping.

## Methods

### Plant materials and growth conditions

A barley DH population consisting of 108 lines from a cross between CM72 (salt-tolerant) and Gairdner (salt-sensitive) were used. Seeds of these lines, the two parental cultivars and two reference cultivars (Yerong (salt-tolerant) and Franklin (salt-sensitive)) were conducted at the Hawkesbury Campus of Western Sydney University, Australia. Seeds (5 per pot) were germinated and grown in 4 L pots containing potting mix augmented with 5 g Osmocoat® slow release fertiliser (Debco Pty Ltd, Victoria, Australia). Two parallel trials were conducted in two glasshouse rooms with grow lamps (600 W) at a temperature of 25 ± 1 °C, 65% relative humidity (RH) and a light/dark photoperiod of 12/12 h. Prior to treatment with NaCl, all plants were watered twice weekly and fertilised with Hoagland’s solution. The plants were subjected to NaCl treatment beginning at Week 5 after sowing at a rate of 50 mM NaCl per day over four consecutive days delivering a stepped final concentration of 200 mM NaCl in an attempt to avoid osmotic shock. All leached salt was collected in a saucer under the pot and re-applied to ensure the stability of concentrations across all treatment pots. Control plants were watered every day. The soil electrical conductivity (EC) was measured regularly with a portable EC meter (HI 991301, HANNA Instruments, Italy). Four weeks after salt treatment, gas exchange and stomatal assays were conducted. Grain yield and biomass were determined at Week 20. In addition, three glasshouse trials evaluating the salinity tolerance of the CM72/Gairdner DH population were conducted at Launceston, Tasmania, Australia. Plant growth conditions and salt treatment were similar to those previously described [35].

### Gas exchange measurements

Gas exchange measurements were made according to O’Carrigan et al. [36] to determine net photosynthetic rate, intercellular CO_2_ concentration, stomatal conductance, transpiration rate, leaf vapour pressure deficit and leaf temperature. Measurements were taken over six consecutive days using an infrared gas analyser (model LI-6400XT, Li-Cor Inc., Lincoln, NE, USA), using the third fully-expanded leaves of seedlings four weeks after the salt treatment ended. The measuring chambers had an air flow rate of 500 mol s^−1^, saturating photosynthetically active radiation (PAR) of 1500 μmol m^−2^ s^−1^, a CO_2_ concentration of 400 μmol mol^−1^ and relative humidity of 65%. Gas exchange measurements were taken at the same time (approximately 10 a.m. to 4 p.m.) as those for stomatal assays.

### Measurement of stomatal parameters

Twelve stomatal traits were analysed as described by Liu et al. [26], Mak et al. [37] and O’Carrigan et al. [36]. The parameters were aperture length (AL), aperture width (AW), aperture width/length (AWL), stomatal pore area (SA), guard cell length (GCL), guard cell width (GCW), guard cell volume (GCV), subsidiary cell length (SCL), subsidiary cell width (SCW), subsidiary cell volume (SCV), stomatal density (SD) and stomatal index (SI). For these measurements, the third fully expanded leaves were collected from the glasshouse and placed on tissue paper soaked in a stabilising solution (50 mM KCl, 5 mM Na^+^-MES, pH 6.1) in Petri dishes. Abaxial epidermal strips were then peeled and mounted on slides using a measuring solution (10 mM KCl, 5 mM Ca^2+^-MES, pH 6.1). Quick peeling and mounting was important to ensure stomatal images were true representations of the stomata found naturally on the whole plant in the glasshouse. Stomatal imaging was conducted using a CCD camera (NIS-F1 Nikon, Tokyo, Japan) attached to a microscope (Leica Microsystems AG, Solms, Germany). All images were analysed using Nikon NIS Element imaging software (Nikon, Tokyo, Japan) and measured with Image J software (NIH, USA).

### Salinity tolerance score

Salt tolerance score was assessed at the seedling stage by combining scores for leaf chlorosis and plant survival (0 = no damage and 10 = all dead) when the most susceptible lines showed severe symptoms [38]. In this study, salt tolerance evaluation was based on the average value from results obtained in 2010, 2014 and 2015 in Launceston, Australia.

### QTLs and statistical analysis

The data regarding photosynthetic and stomatal traits, biomass and grain yield, measured under control and saline conditions, were used for QTL analysis. The ratios of these traits in saline to control conditions were also tested for QTL identification. A genetic linkage map for this population was constructed using 886 markers including 868 Diversity Array Technology (DArT) and 18 Simple Sequence Repeat (SSR) markers. The software package, MapQTL 6.0 [39], was used to detect QTLs. QTLs were first analysed by interval mapping (IM). Following this, the closest marker at each putative QTL identified with interval mapping was selected as a cofactor, and the selected markers were used as genetic background controls in the approximate multiple QTL model (MQM). Logarithm of the odds (LOD) threshold values, applied to declare the presence of a QTL, were estimated by performing genome-wide permutation tests implemented in MapQTL version 6.0 using 1000 permutations of the original data set for each trait, resulting in a 95% LOD threshold of around 3.0. To determine the effects of other traits on the QTLs for salinity tolerance, the QTLs for salinity tolerance were re-analysed using other traits as covariates. Two LOD support intervals around each QTLs were established, by taking the two positions, left and right of the peak, that had LOD values of two less than the maximum [39], after performing restricted MQM mapping which does not use markers close to the QTL. The percentage of variance explained by each QTL (R^2^) was obtained using restricted MQM mapping implemented with MapQTL 6.0. Graphical representation of linkage groups and QTLs was carried out using MapChart 2.2 [40]. Frequency distribution analysis was performed using SigmaPlot 12 (Systat Software Inc., San Jose, CA, USA). Skewness analysis was conducted. If skewness is less than −1 or greater than +1, the distribution is highly skewed; if skewness is between −1 and − ½ or between + ½ and +1, the distribution is moderately skewed, and if skewness is between − ½ and + ½, the distribution is approximately symmetric [41].

### Genomic analysis of potential genes for salinity tolerance

The sequence marker, Bmac0209, associated with the QTL for salinity tolerance score on 3H was used to identify candidate genes for salinity tolerance. The genome sequence of this region was retrieved by following a BLAST search (http://webblast.ipk-gatersleben.de/barley/). A morex_contig, 84335, was found to be homologous with Bmac0209. The physical map position of this contig was located at 51.77 cM on 3H. Barley genomic data and gene annotations were downloaded from ftp://ftpmips.helmholtz-muenchen.de/plants/barley/public_data/ [42] and ftp://ftpmips.helmholtz-muenchen.de/plants/barley/public_data/popseq_IPK/ [43]. Annotated genes between 46.74 and 56.72 cM were deemed to be potential genes for salinity tolerance (Additional file 1: Table S1).

## Results

### The DH and parental lines show a large diversity in salinity tolerance

**Fig. 1:**
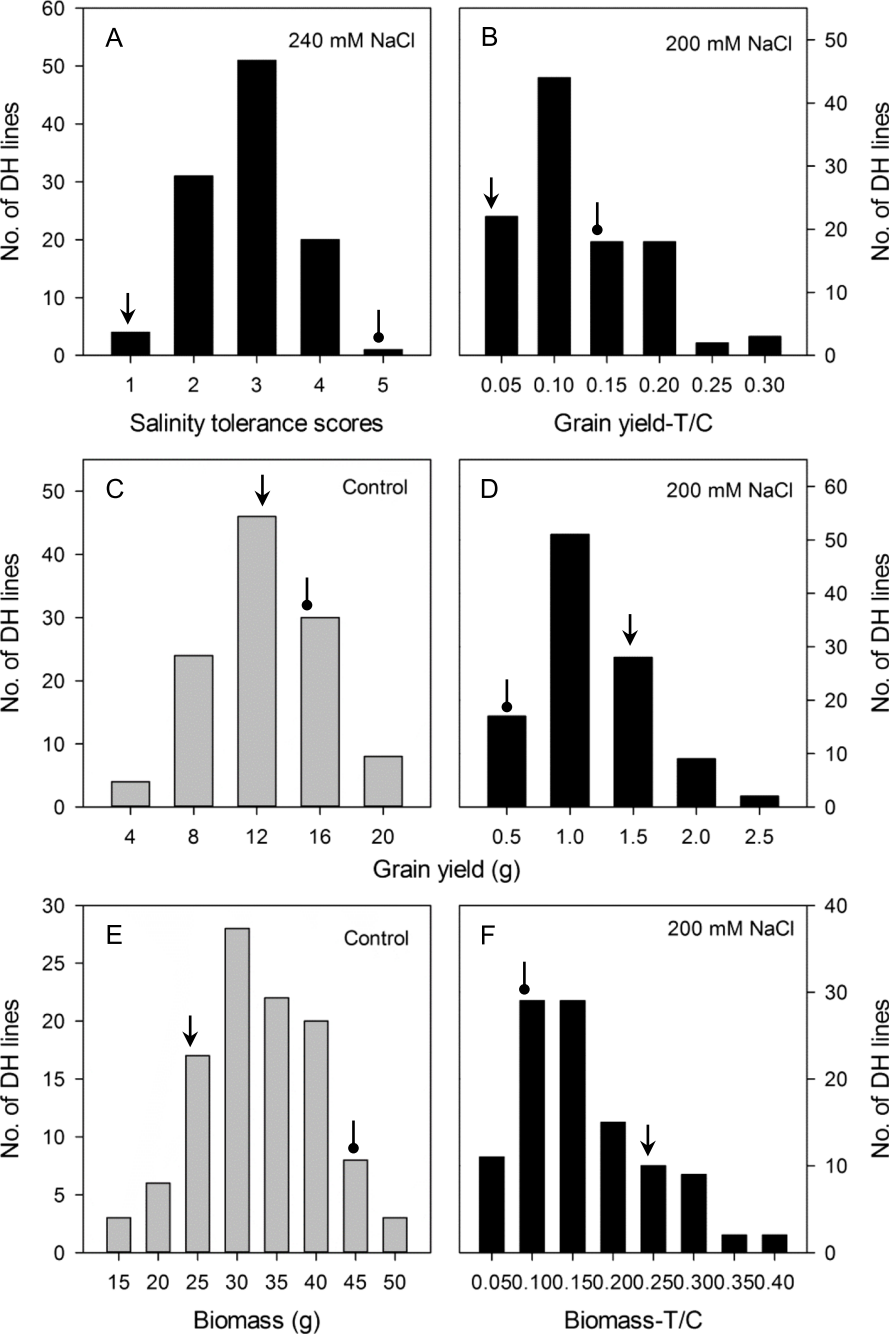
Frequency distribution for salinity tolerance score (**a**), relative grain yield-T/C (**b**), grain yield (**c**, **d**), biomass (**e**) and relative biomass-T/C (**f**) of DH lines derived from the cross of CM72 and Gairdner under control and salt treatment. T/C: the ratio of traits under salt treatment (T) and control (C). *Arrow* represents CM72 and *oval arrow* represents Gairdner. Data are averages of four replicates. Salinity tolerance score are averaged over three years with three replicates each year

**Fig. 2:**
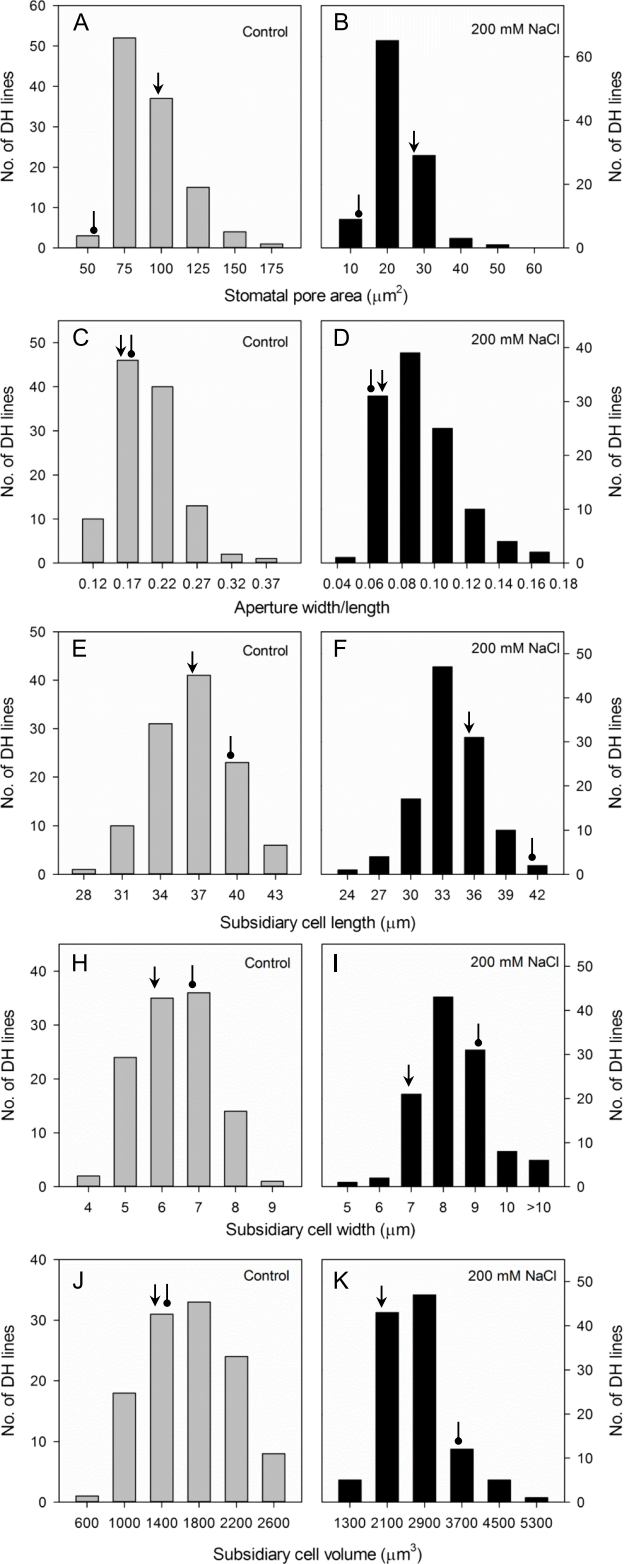
Frequency distribution of stomatal traits in control and salt treatment. Shown are stomatal pore area (**a**, **b**), aperture width/length (**c**, **d**), subsidiary cell length (**e**, **f**), subsidiary cell width (**h**, **i**) and subsidiary cell volume (**j**, **k**) of DH lines derived from the cross of CM72 and Gairdner, under control and salinity treatment conditions. *Arrow* represents CM72 and *oval arrow* represents Gairdner. Data are averages of 16–73 cells from 4 replicates

**Fig. 3:**
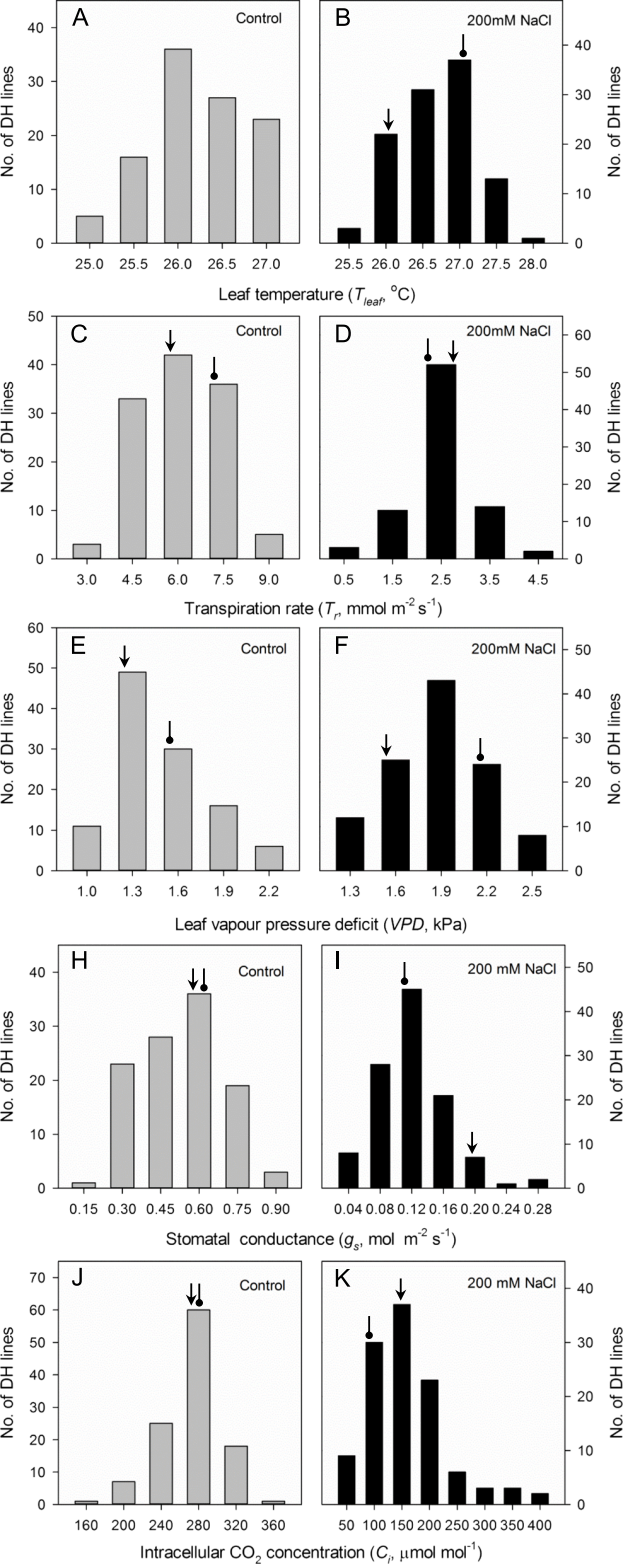
Frequency distribution of gas exchange traits in control and salt treatment. Shown are leaf temperature (**a**, **b**), transpiration rate (**c**, **d**), leaf vapour pressure deficit (**e**, **f**), stomatal conductance (**h**, **i**) and intracellular CO2 concentration (**j**, **k**) of DH lines derived from the cross of CM72 and Gairdner. *Arrow* represents CM72 and *oval arrow* are Gairdner. Data are averages of four replicates

**Fig. 4:**
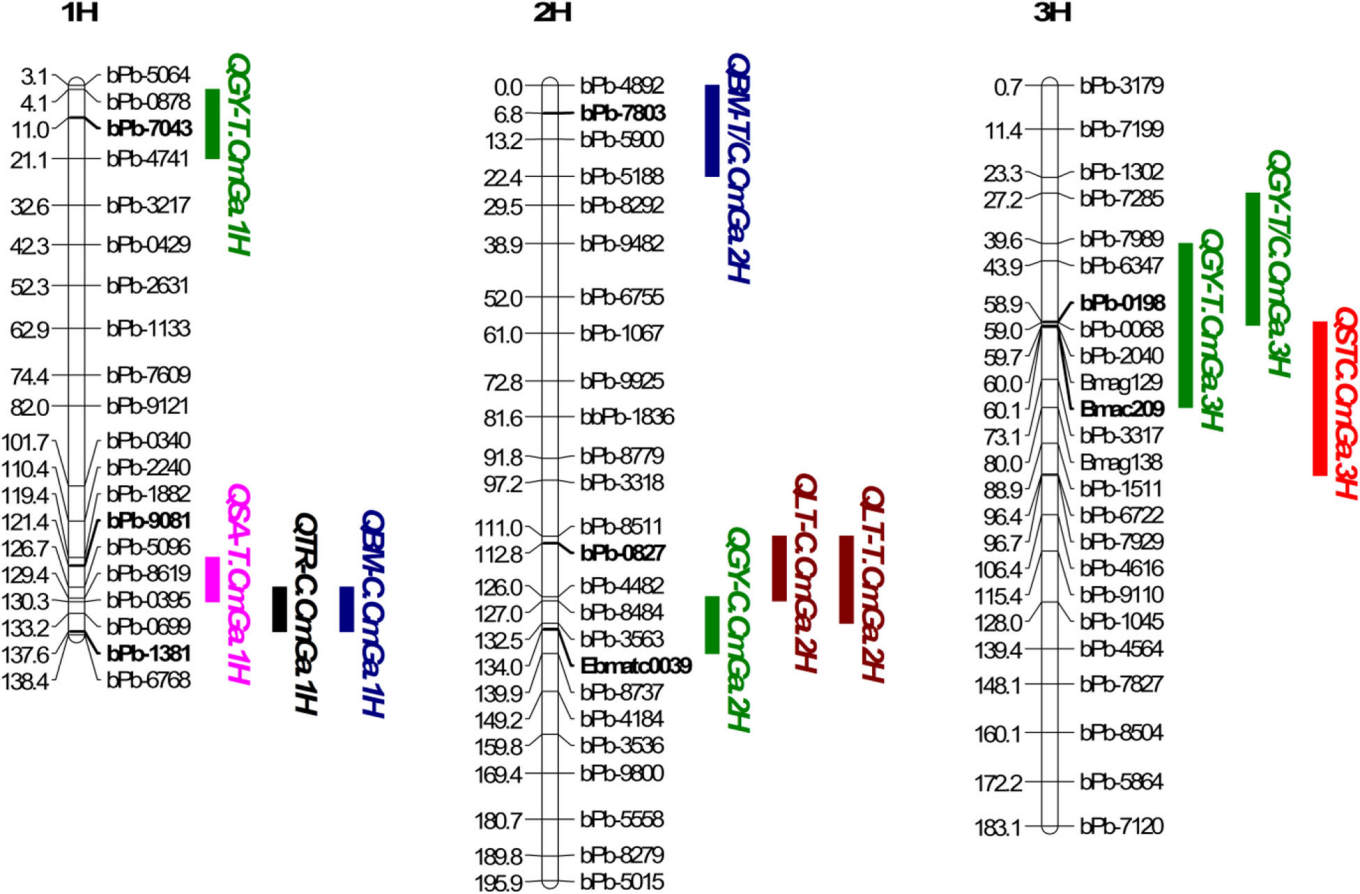
QTLs associated with salinity tolerance (in *red*), grain yield (in *green*), biomass (in *blue*), stomatal area (in *pink*), transpiration rate (in *black*) and temperature of leaves (in *brown*). For clarity, only part of the chromosome regions which cover 2-LOD interval of all the QTLs are shown. C: Control; T: salt treatment

Significant differences in stomatal and photosynthetic traits between parental line CM72 and Gairdner were described in Liu et al. [26]. The 108 DH lines showed significant differences in salinity tolerance, with CM72 being scored as 1 and Gairdner at 5 (Fig. 1a). Grain yield, biomass, leaf temperature, transpiration rate, stomatal area and other parameters of the DH lines under control or saline conditions displayed continuous frequency distributions (Figs. 1, 2 and 3). Of all 26 traits listed in Figs. 1, 2 and 3, 17 showed approximately symmetric distribution, six moderately skewed and three highly skewed (Additional file 2: Table S2). Salinity stress caused a significant shift in the distribution of photosynthetic and stomatal traits and in grain yields (Figs. 1, 2 and 3). Stomatal conductance, transpiration rate and intracellular CO_2_ concentration showed a distribution skewed to lower values under salt stress, whereas leaf vapour pressure deficit and leaf temperature displayed a distribution skewed to higher ranges (Fig. 3). For stomatal traits, stomatal pore area, aperture width/length and subsidiary cell length had distributions skewed to lower values under salt stress; in contrast, subsidiary cell width and subsidiary cell volumes showed a distribution skewed to higher ranges (Fig. 2). Grain yields were skewed to lower values under salinity treatment. Of the two parental lines, CM72 showed better performance than Gairdner for all traits under saline stress (Figs. 1, 2 and 3). This enabled the QTL mapping to identify a total of 11 QTLs (Fig. 4, Table 1 and Additional file 3: Figure S1 with LOD values of > 3.0 Additional file 4: Figure S2).

**Table 1:** QTL and tentative QTL identified for different traits under saline and control conditions in a double haploid population derived from a cross between CM72 and Gairdner

Trait	QTL	Linkage group	Position	LOD	R^2^ (%)	Nearest marker
GY-T	QGY-T.CmGa.1H	1H	11	3.95	12.5	bPb-7043
SA-T	QSA-T.CmGa.1H	1H	121.4	3.12	12.5	bPb-9081
TR-C	QTR-C.CmGa.1H	1H	137.6	3.52	14	bPb-1381
BM-C	QBM-C.CmGa.1H	1H	137.6	3.79	15	bPb-1381
GS-C		1H	137.6	2.76	11.2	bPb-1381
CI-C		1H	137.6	2.75	11.2	bPb-1381
BM-T/C	QBM-T.CmGa.2H	2H	6.8	3.49	14	bPb-7803
TR-T/C		2H	6.8	2.54	10.4	bPb-7803
GS-T		2H	10.3	2.54	10.3	bPb-1949
LT-C	QLT-C.CmGa.2H	2H	112.8	4.19	16.5	bPb-0827
LT-T	QLT-T.CmGa.2H	2H	112.8	3.09	11.2	bPb-0827
GY-C	QGY-C.CmGa.2H	2H	134	3.77	15	Ebmatc0039
VPD-T/C		2H	153.6	2.63	10.7	bPb-2701
GY-T	QGY-T.CmGa.3H	3H	58.9	4.35	13.9	bPb-0198
GY-T/C	QGY-T/C.CmGa.3H	3H	58.9	4.42	17.3	bPb-0198
STC	QSTC.CmGa.3H	3H	60.1	4.29	16.8	Bmac209
AWL-T		3H	136.9	2.78	11.3	bPb-3634
SCL-T		4H	122.5	2.98	12	bPb-6153
LT-T		5H	53.3	2.69	9.7	bPb-2762
SCW-C		6H	93.5	2.81	11.4	EBmac602
SCV-C		6H	93.5	2.58	10.5	EBmac602
LT-C		7H	63.9	2.81	9.5	bPb-1209

Tentative QTL: 2.5 < LOD < 3.0. QTL: LOD > 3.0

Abbreviations: *−C* traits under control conditions, *−T* traits under salt treatment, *−T/C* the ratio of traits under salt treatment to traits under control conditions, *GY* grain yield, *BM* biomass, *STC* salinity tolerance score, gas exchange traits, *TR* transpiration rate, *LT* leaf temperature, *GS* stomatal conductance, *CI* intercellular CO_2_ concentration, *VPD* leaf vapour pressure deficit, *SA* stomatal pore area, *AWL* aperture width/length, *SCL* subsidiary cell length, *SCW* subsidiary cell width, *SCV* subsidiary cell volume

### Significant QTLs for gas exchange and stomatal traits under control and saline conditions

One QTL for transpiration rate under control conditions (QTR-C.CmGa.1H) was detected on chromosome 1H close to bpb-1381 and explained 14% of the phenotypic variation. Two QTLs associated with leaf temperature were identified at the same position on chromosome 2H, QLT-C.CmGa.2H under control conditions and QLT-T.CmGa.2H in the salinity treatment, respectively explaining 16.5 and 11.2% of the phenotypic variation (Fig. 4, Table 1 and Additional file 4: Figure S2). Only one significant QTL, QSA-T.CmGa.1H associated with stomatal pore area under salinity treatment, was found on the long arm of chromosome 1H; this had a LOD value of 3.12. This QTL, with the closest marker being bPb-9081, accounted for 12.5% of the phenotypic variation (Fig. 4, Table 1 and Additional file 4: Figure S2).

### Significant QTLs for grain yield, biomass and salinity tolerance under control and saline conditions

Four QTLs for grain yield were found on chromosomes 1H, 2H and 3H: QGY-T.CmGa.1H and QGY-T.CmGa.3H under salinity treatment; QGY-C.CmGa.2H under control conditions; and QGY-T/C.CmGa.3H based on the ratio of salinity stress relative to control. QGY-C.CmGa.2H (with Ebmatc0039 as its nearest marker) explained 15.0% of the phenotypic variation and QGY-T.CmGa.1H (with bPb-7043 as its closest marker) accounted for 13.9% of the phenotypic variation (Fig. 4, and Table 1). In addition, two QTLs for biomass were identified on chromosomes 1H and 2H. The QTL, QBM-C.CmGa.1H, found under control conditions and flanked by bPb-1381, explained 15% of the phenotypic variation. In contrast, QBM-T.CmGa.2H, found under salinity treatment and with the nearest marker being bPb-7803, is located near the telomere of the short arm of chromosome 2H and explained 14% of the phenotypic variation (Fig. 4 and Table 1). Furthermore, QST.CmGa.3H (60.1 cM), which is associated with salinity tolerance, was identified on chromosome 3H with Bmac209 being the nearest marker. This QTL explained 16.8% of phenotypic variation and had a LOD value of 4.29 (Fig. 4 and Table 1).

### Tentative QTL for gas exchange traits under control and saline conditions

Apart from the significant QTLs, 11 tentative QTLs (2.5 < LOD < 3.0) were also identified in this study (Additional file 3: Figure S1 and Table 1). A QTL (GS-C) for stomatal conductance and one for intercellular CO_2_ concentration (CI-C) under control conditions were located on chromosome 1H near marker bPb-1381 (Additional file 3: Figure S1 and Table 1). There are three tentative QTLs on chromosome 2H that are associated with stomatal conductance under saline conditions (GS-T), transpiration rate (TR-T/C) and vapour pressure deficit (VPD-T/C) found under salinity stress relative to control (Additional file 3: Figure S1 and Table 1). Also, a QTL for leaf temperature under salt treatment (LT-T) and one (LT-C) under control conditions were located on chromosomes 5H and 7H, respectively (Additional file 3: Figure S1 and Table 1). In addition, four tentative QTL were identified for stomatal traits. One QTL for aperture width/length under salt treatment (AWL-T) was found on chromosome 3H (Additional file 3: Figure S1 and Table 1). Another QTL for subsidiary cell length under salt treatment (SCL-T) was identified on chromosome 4H, while QTLs for subsidiary cell width (SCW-C) and subsidiary cell volume (SCV-C) under control conditions were both found on chromosome 6H (Additional file 3: Figure S1 and Table 1).

### Co-localisation of phenotypic traits

**Fig. 5:**
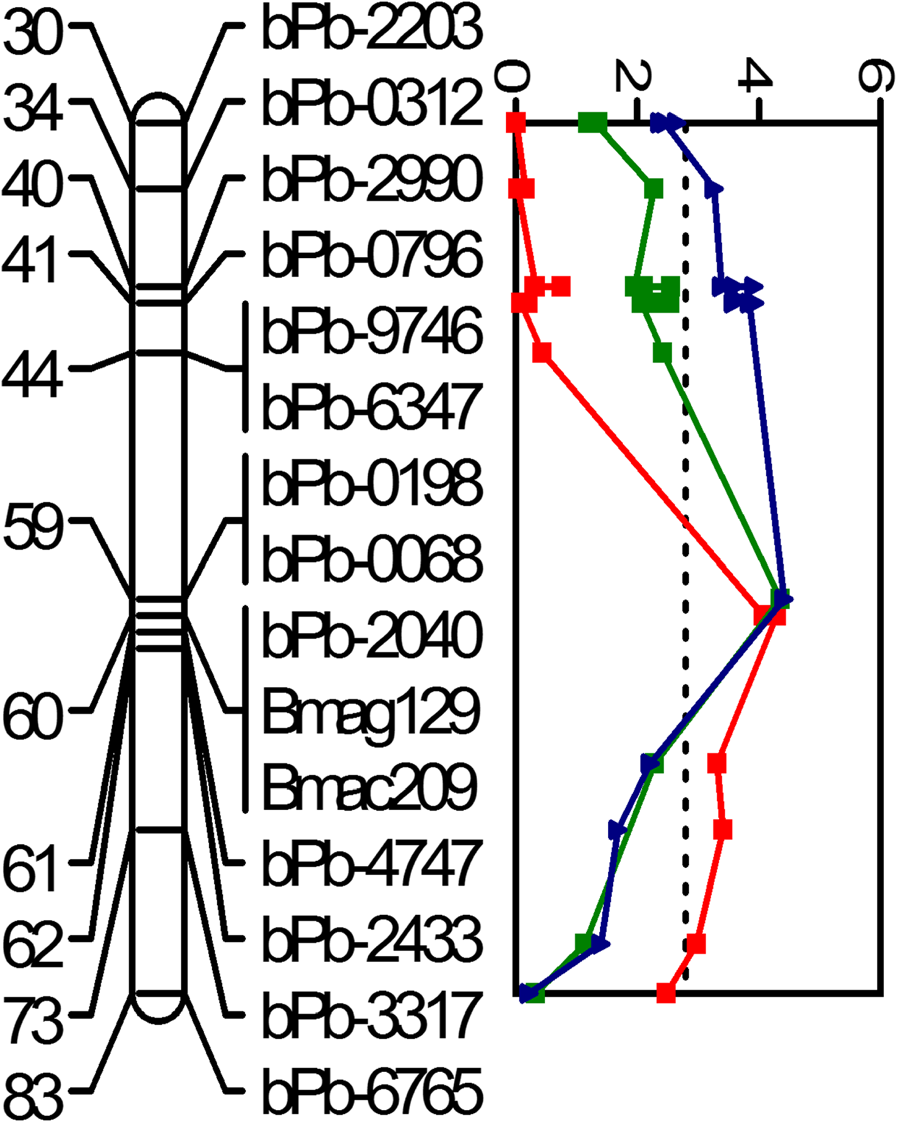
QTL associated with salinity tolerance (*red solid line*), grain yield-T (*green solid line*) and grain yield-T/C (*blue solid line*) located at similar position on 3H. The dotted line around LOD 3.0 is a line of significance. T: salt treatment; T/C: the ratio of traits under salt treatment (T) and control (C)

There are five clusters of QTLs for different traits (Fig 4 and Additional file 3: Figure S1). On chromosome 1H, QTLs for transpiration rate (TR-C), biomass (BM-C), stomatal conductance (GS-C) and intercellular CO_2_ concentration (CI-C) under control conditions were located at the same position (137.6 cM) and shared a common nearest marker, bpb-1381 (Table 1). On chromosome 2H, two QTLs for biomass and transpiration rate relative to control (Bm-T/C, TR-T/C) were both at 6.8 cM with bpb-7803 being the closest marker, while another QTL for stomatal conductance under salt treatment (GS-T) was located at 10.3 cM close to QBM-T/C.CmGa.2H and QTR-T/C.CmGa.2H (Table 1). In addition, QTLs for leaf temperature under control (LT-C) and saline conditions (LT-T) were at same position (112.8 cM) on chromosome 2H with bpb-0827 as the closest marker (Table 1). Furthermore, QTLs for grain yield (GY-T, GY-T/C) on chromosome 3H (58.9 cM) were located close to a QTL for salinity tolerance score (60.1 cM) (Fig. 5 and Table 1). Also, QTLs for subsidiary cell width (SCW-C) and subsidiary cell volume (SCV-C) under control conditions were both at 93.5 cM on chromosome 6H with EBmac602 as the nearest marker (Table 1).

### Identification of candidate genes for salinity tolerance on 3H

QTLs for grain yield relative to the control were located close to the QTL for salinity tolerance score on chromosome 3H (Figs. 4 and 5; Table 1). This led to further investigation of the possible genes controlling salinity tolerance in barley. Annotated genes close to SSR marker, Bmac0209, were examined from the published sequence of the barley genome. There is a number of potential salt tolerance-related genes within this region of interest (Additional file 1: Table S1). These genes related to: reactive oxygen species (ROS) detoxification, including a peroxidase (AK249079.1 at 51.63 cM) and a respiratory burst oxidase-like protein (MLOC_81745.1 at 50.67 cM); ion transport, including a potassium channel (MLOC_74879.2 at 51.27 cM), an outward rectifying potassium channel (MLOC_18521.1 at 55.1 cM), a vacuolar cation/proton exchanger (MLOC_13658.1 at 51.35 cM), a V-type proton ATPase (AK251977.1 at 51.63 cM), a voltage-gated chloride channel (MLOC_57123.4 at 56.44 cM); and some transcription factors possibly involved in guard cell signal transduction such as Myb domain protein MYB (MLOC_7981.1 at 51.63 cM), WD-40 repeat protein (AK370701 at 51.63 cM), Ca^2+^ dependent protein kinase CDPK (MLOC_12765.1 at 47.04 cM), Ca^2+^ independent protein kinase CIPK (MLOC_9827.2 at 49.29 cM) and calcineurin-B like activator CBL (MLOC_60474.3 at 55.17 cM).

## Discussion and conclusion

### Intercellular CO_2_ concentration, transpiration rate and stomatal conductance are genetically linked to biomass production in barley

Salinity tolerance in plants including barley is inherently complex, controlled by polygenic traits and is affected by various mechanisms influencing photosynthesis [44–46]. Under control conditions, QTLs for intercellular CO_2_ concentration (CI-C), transpiration rate (TR-C) and stomatal conductance (GS-C) were closely located together with that for biomass (BM-C) on chromosome 1H (Additional file 3: Figure S1 and Table 1). QTLs associated with photosynthetic traits have rarely been reported in barley due to measurement procedures and the complicated, dynamic processes of these phenotypic traits. Liu et al. [33] identified several QTLs located on chromosomes 2H, 3H and 7H associated with intercellular CO_2_ concentration, transpiration rate and stomatal conductance from barley flag leaves on plants grown under normal conditions. QTLs for net photosynthetic rate, transpiration rate and stomatal conductance have also been found in rice [29]. Therefore, photosynthetic parameters have the potential to indicate salinity tolerance in grasses [47]. In our experiment, co-localisation of QTLs for gas exchange traits with those for biomass production demonstrated that intercellular CO_2_ concentration, transpiration rate and stomatal conductance are genetically linked to biomass production in barley. Moreover, QTLs for relative transpiration rate (TR-T/C) and relative biomass (BM-T/C) contributing to growth and yield under salt stress were identified at the same position on chromosome 2H (Additional file 3: Figure S1 and Table 1).

NaCl-induced accumulation of ABA in leaves leads to stomatal closure and reduced transpiration rate, thereby contributing to increased water use efficiency in plants [8]. Initial stomatal closure can serve as a rapid and effective response to salinity; however, long-term stomatal closure will limit CO_2_ uptake, photosynthesis and plant growth [27]. CO_2_ and water availability strongly influence stomatal opening and closure. Stomatal opening or closure directly affect stomatal conductance which further influences CO_2_ intake and transpirational water loss [7]. Short-term, elevated CO_2_ concentrations provoke stomatal closure, whereas long-term, elevated CO_2_ concentrations decrease stomatal density leading to reductions in transpiration [7, 48]. Stomatal conductance significantly influences net photosynthetic rate and is one of the key parameters limiting photosynthesis in barley [49]. In our study, we found genetic evidence for the importance of photosynthetic traits for barley production under salinity stress. Therefore, promoting leaf photosynthetic capacity and genetic modification of traits on which photosynthesis relies, are important approaches to enhancing crop biomass [50].

### Linkage between gas exchange and stomatal traits under salinity stress

Gas exchange characteristics are influenced by stomatal structure, aperture and density [10]. Salt tolerance was associated with lower stomatal density and decreased stomatal area in *Chenopodium quinoa* [18], and a positive correlation between stomatal frequency and transpiration rate was reported in barley [51]. In rice, it was reported that high stomatal density was associated with high photosynthetic rate in *Indica* cultivars, while *Japonica* cultivars had higher transpiration efficiency [52]. In addition, it has been suggested that salinity may have a relatively direct impact on the photosynthetic apparatus independent of that on stomata [53]. Moreover, a remarkably negative correlation between stomatal density and size was found in lowland rice but no common QTL was found for these traits [8]. One of the aims of this study was to find potential QTLs connecting stomatal and photosynthetic traits for salinity tolerance in barley. In this study, a stomatal trait QTL for stomatal pore area (SA) was associated with the gas exchange traits of, transpiration rate (TR), stomatal conductance (GS) and intercellular CO_2_ concentration (CI) (Additional file 3: Figure S1). Conversely, the remaining stomatal trait QTLs for aperture width/length (AWL) and subsidiary cell length (SCL) did not show links with the gas exchange traits QTLs for leaf temperature (LT) and vapour pressure deficit (VPD). Therefore, our findings suggest that stomatal traits and gas exchange traits are not genetically well-linked. One potential reason could be that gas exchange measurements determine leaf CO_2_ assimilation and H_2_O transpiration, which are governed by stomatal and non-stomatal factors. However, the stomatal traits measured in this study may have little influence over the non-stomatal factors such as the genetic control of photosynthetic machinery [9, 10, 48].

### QTLs and candidate genes for salinity tolerance score and grain yield

Salinity tolerance score, assessed through the combination of plant survival and leaf wilting, has been used for evaluating barley salt tolerance in our previous studies [35, 38, 54]. Crop yield under salinity stress is a result of balancing resource allocation between growth and defence against stress, since responding to stress is deleterious to growth and yield [55]; therefore, the ability to produce high grain yield in saline soils is the ultimate criterion of salinity tolerance. In this study, we identified one QTL controlling salt tolerance score (QST.CmGa.3H) using plant survival and leaf wilting as an evaluation index. This QTL, located at 60.1 cM on chromosome 3H (with Bmac209 as the nearest marker) explained 16.8% phenotypic variation. Interestingly, the QTL for grain yield relative to the control (QGY-T/C.CmGa.3H) was only 1.2 cM away from this QTL for salinity tolerance score (Fig. 1). Therefore, we attempted to identify candidate genes for salinity tolerance on chromosome 3H using the published sequence of the barley genome (Additional file 1: Table S1). These candidate genes included those involved in ROS detoxification, photosynthesis, ion transport and signal transduction. Plants can perceive stress through transmembrane osmo-receptors and transduce the perception of environmental stimuli via internal signalling pathways. Induced transcription factors (TFs) and post-translational regulation of TFs lead to the expression of functional, downstream response genes associated with ion channels, secondary metabolite biosynthesis, ROS detoxification, stomatal closure, growth regulation, cell death as well as those encoding Late Embryogenesis Abundant (LEA) proteins [55]. Although we cannot rule out other genes in this region, these are candidates for further fine-mapping and functional analysis to verify their roles in salt tolerance in barley. Near isogenic lines are being developed in our current research work for fine mapping of these candidate genes.

### Co-localisation of QTLs associated with salinity tolerance

Using various genetic populations, some QTLs for salinity tolerance in barley have been found and are associated with chlorophyll content, chlorophyll fluorescence, proline content, water soluble carbohydrates, relative water content [56], ion content [57, 58], and salinity tolerance [35, 38, 54, 59]. Previous QTL studies of barley salinity tolerance have lacked information on the genetic mechanisms underlying stomatal traits and gas exchange parameters. Interestingly, many QTLs for stomatal traits and gas exchange parameters (Figs. 2 and 3; Additional file 2: Table S2) co-localised with previously identified QTLs for agronomic or physiological traits (Fig. 4, Table 1 and Additional file 4: Figure S2). The co-localization of QTLs for stomatal conductance, leaf vapour pressure deficit, leaf temperature, chlorophyll content, chlorophyll fluorescence, water soluble carbohydrate and relative water content with salinity tolerance is not unexpected, because these traits are associated with photosynthesis and transpiration in barley. The relationship among these traits indicates that these parameters may not be independent but interacting. They may be co-regulated for the protection of photosynthetic apparatus, an important factor in tolerance to salinity stress [2]. In addition, the co-localisation of QTLs for stomatal conductance and leaf temperature under control or saline conditions could be linked with genes whose proteins control K^+^, Na^+^ and Cl^−^ homeostasis. Stomatal opening or closure is controlled by guard cells and adjacent subsidiary cells, and the ‘shuttling’ of ions and solutes between the two cell types [60] using channels and transporters to maintain ionic homoeostasis in these cells. The shuttle transport of K^+^ between subsidiary and guard cells, resulting in prompt stomatal opening and closure. K^+^ accumulation was generally detected in subsidiary cells during stomatal closure [60, 61]. K^+^ inward and outward rectifying channels and slow anion channels in guard cell could be responsive to ion shuttle transport within the stomatal complex [61–64]. Interestingly, six genes from the published barley genome sequence related with the transport of K^+^ and anions are located close to QTL, QGY-T/C.CmGa.3H and to the SSR marker, Bmac0209 (Additional file 1: Table S1). Therefore, the fine mapping of genes encoding K^+^ and Cl^−^ channels should be performed followed by their functional analysis to verify their roles in salinity tolerance in barley.

## Additional files

Additional file 1: Table S1. List of candidate genes within 10 cM around Bmac0209 on chromosome 3H of barley. (XLSX 37 kb)
Additional file 2: Table S2. Skewness analysis of average data of all phenotypic traits from the DH lines used for QTL mapping in this study. (XLSX 106 kb)
Additional file 3: Figure S1. QTLs associated with different traits of the DH lines derived from the cross of CM72 and Gairdner under control and salinity conditions. (PPTX 429 kb)
Additional file 4: Figure S2. Distribution of QTLs discovered in this study (LOD value > 3.0). (PPTX 124 kb)

## Abbreviations

AL: Aperture length
AW: Aperture width
AWL: Aperture width/length
CI: Intercellular CO_2_ concentration
DArT: Diversity Array Technology
DH: Double haploid
GCL: Guard cell length
GCV: Guard cell volume
GCW: Guard cell width
GS: Stomatal conductance
IM: Interval mapping
LEA: Late Embryogenesis MBRYOGENESIS Abundant
LOD: Logarithm of the odds
LT: Leaf temperature
MQM: Multiple QTL model
PAR: Photosynthetically active radiation
QTLs: Quantitative trait loci
ROS: Reactive oxygen species
SA: Stomatal pore area
SCL: Subsidiary cell length
SCV: Subsidiary cell volume
SCW: Subsidiary cell width
SD: Stomatal density
SI: Stomatal index
SSR: Simple sequence repeat
TFs: Transcription factors
TR: Transpiration rate
VPD: Vapour pressure deficit
