# Supplementary material for: QTLs for stomatal and photosynthetic traits related to salinity tolerance in barley
Source: BMC Genomics. 2017 Jan 3;18:9. doi: 10.1186/s12864-016-3380-0 (PMC5210286; doi:10.1186/s12864-016-3380-0)
Supplement: Additional file 3: Figure S1. — QTLs associated with different traits of the DH lines derived from the cross of CM72 and Gairdner under control and salinity conditions. (PPTX 429 kb) [file 12864_2016_3380_MOESM3_ESM.pptx]

## Slide 1
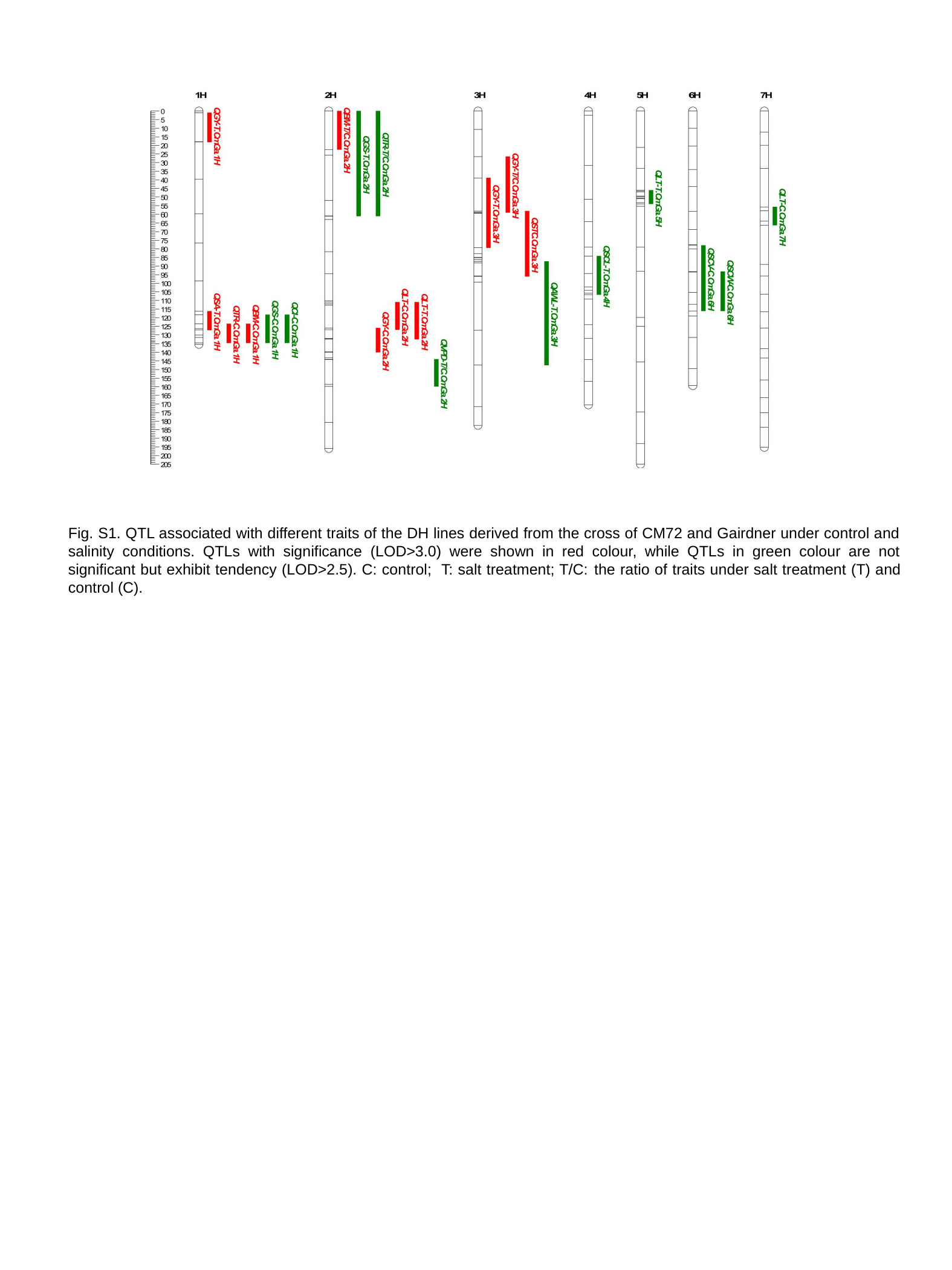

# Fig. S1. QTL associated with different traits of the DH lines derived from the cross of CM72 and Gairdner under control and salinity conditions. QTLs with significance (LOD>3.0) were shown in red colour, while QTLs in green colour are not significant but exhibit tendency (LOD>2.5). C: control; T: salt treatment; T/C: the ratio of traits under salt treatment (T) and control (C).
